# Total starch in animal feeds and silages based on the chromatographic determination of glucose

**DOI:** 10.1016/j.mex.2018.01.009

**Published:** 2018-01-31

**Authors:** Marcela María Salazar Murillo, Fabio Granados-Chinchilla

**Affiliations:** a Sede de Occidente, Universidad de Costa Rica, Recinto de Grecia, Tacares, 118, 20305, Alajuela, Grecia, Costa Rica; b Centro de Investigación en Nutrición Animal (CINA), Universidad de Costa Rica, 11501-2060, Ciudad Universitaria Rodrigo Facio, San José, Costa Rica

**Keywords:** Starch in animal feed and silage, Starch, Glucose, Enzymatic hydrolysis, Liquid chromatography, Refractive index detector, Feed, Silage

## Abstract

Starch is an important nutrient in animal feed, and so its analysis is of considerable concern as it is one of the most relevant energy containing fractions. Method AOAC 996.11 was modified to exchange the enzymometric and colorimetric step full approach to a simpler HPLC amine-based column one. The method was optimized and validated for its application in animal feeds and silages.

• We demonstrated that the method could be used for quality control for animal feeds and silages
• We modified the final incubation time, the initial sample mass, the quantity of enzyme added and buffered, to pH 6.2, the medium to which α-amylase is added.
• We applied a chromatographic analysis of the glucose that resulted from starch enzymatic hydrolysis, via a refractive index detector and amine-based chromatographic column.

## Graphical abstract

**Figure:**
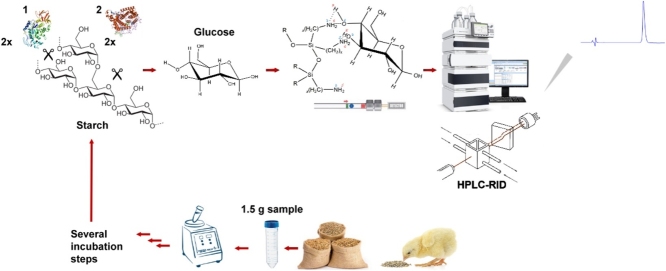

## Method details

### Background

Starch, a frequently analyzed component of animal feedstuffs is incorporated into production animals diets such as beef and dairy cattle [1], chickens [2], swine [3] as a primary source of energy and to improve production. It is also a primary nutrient in some formulas of pet compound food (both dried and canned) [4,5]. Starch concentrations in feed grains range from 40 g/100 g, in oats, up to 80 g/100 g in rice (both in dry matter basis), depending on variety, location, climatic conditions, and agronomic practices [6]. On the other hand, silages (e.g., whole plant maize silage) are reserved for ruminant rations; starch content ranges from ca. 20 g/100 g to 60 g/100 g of dry matter, variations resulting in plant maturity when harvested [7].

Despite its relevance, few methods are available for the determination of total starch present in the feed. Only one method is currently available as an AOAC Official Method^SM^ (i.e., 920.40 for starch in the feed). This last essay is based on direct acid hydrolysis which is time-consuming and, depending on the compound feed ingredients, may lack specificity. Other quantification approaches include polarimetry [Ewers, ISO 6493:2000], Megazyme kit, YSI analyzer and NIR [ISO 12099:2010]. Hall described a dietary Starch in Animal Feeds and Pet Food by an Enzymatic-Colorimetric Method [8]. Demonstrating once again the relevance of enzyme assisted sample treatment during feed analysis and analytical chemistry [9]. More recently, an LC/MS approach was developed for estimating reducing sugars in grains during bioethanol production and monitoring [10].

On the other hand, method 996.11 is an enzymatic-colorimetric (i.e., amyloglucosidase/α-amylase) method established to be applied to cereals. As feed mayor ingredients are grains (e.g., corn, wheat, soybean, rice, millet, sorghum, dried distillers grains) products and by-products [11] is reasonable to use this method as a starting point.

Herein we reported the modification, optimization, and validation of method AOAC-AACC 996.11, designed originally for cereals, and it was applied to animal feeds and silages. We substituted the spectrophotometric glucose oxidase and peroxidase-based determination for a more straightforward, less expensive (i.e., we eliminate the purchase, transport, and storage of the glucose-specific enzymes and reagents such as 4-aminoantipyrine, to name a few), and accurate HPLC assay. Furthermore, sample analysis in HPLC when coupled to an automated liquid sampler reduce the analyst involvement during the measurement step. We also performed modifications which improve starch recovery from feed and silage.

### Reagents

Acetonitrile (ACN, chromatographic grade) was purchased from J.T. Baker (Avantor Materials, PA, USA). Amyloglucosidase (from *Aspergillus niger*, ∼120 units/mg, 10113), MOPS (3-(*N*-morpholino)propanesulfonic acid, 99.5%, M1254) and α-amylase (A4551, lyophilized powder, 500–1 500 units/mg protein, 93–100%) were purchased from Sigma-Aldrich (St. Louis, MO, USA).

### In-feed starch enzymatic conversion to glucose

A representative (1.500 ± 0.100) g, previously sieved to 1 mm (using a ZM200 ultracentrifuge mill, Retsch GmbH, Haan, Germany), feed subsample was used for extraction. This sample portion was weighted in centrifuge tubes (50 mL, self-standing, polypropylene, Corning, NY, USA). Afterwards, 10 mL of an aqueous ethanol 80 mL/100 mL solution is added. The mixture was incubated for 10 min at an 80 °C water bath (Thermo Scientific™ Precision™, TS-GP0-5PM, Thermo Fisher Scientific, Inc. Waltham, MA, USA) and later centrifuged (at 2000*g* for 10 min, Thermo Scientific™ Sorvall™ ST 16R Thermo Fisher Scientific, Inc. Waltham, MA, USA). The supernatant was discarded (this step removes soluble reducing sugars originally present in the sample), and the sediment was reserved for further analysis to which 200 μL ethanol solution is added and vortexed (speed 7, Vortex-Genie 2, Scientific Industries, Inc, Bohemia, New York, USA) for 1 min. Then, 3.00 mL of a heat stable α-amylase (previously prepared 3 000 units/mL amylase solution on a MOPS aqueous buffer, adjusted to a final pH of 6.2) were added and vortexed for 1 min. Immediately, the mixture is incubated for 2 min at 80 °C, vortexed for 1 min, returned to the water bath for an additional 3 min incubation and vortexed again for an additional minute. After that, the mixture is incubated for 5 min at 50 °C in a water bath. A 4.00 mL aliquot of a previously prepared acetate aqueous buffer (adjusted to a 4.5 pH, with 0.2 μg mL^−1^ sodium azide) was added conjointly with a 200 μL amyloglucosidase (prepared in the buffer mentioned above, 200 units mL^-1^). The resulting mixture was incubated for 15 h at 50 °C in a water bath. The supernatant was filtered through a Whatman 541 ashless filter by gravity and, then, by pressure through a 0.45 μm filter (used sequentially, Acrodisc^®^ syringe filters with PVDF hydrophilic membrane, Pall Corporation, Port Washington, NY, USA). The filtrate was recovered into a 10 mL volumetric flask (to account for any evaporation suffered during incubation), which is made up to capacity with water. Afterwards, 2 mL are transferred into an HPLC vial for injection (Agilent Technologies, Santa Clara, CA, USA).

### Chromatographic conditions

All assays performed using an Agilent Technologies LC system equipped with 1260 infinity quaternary pump (61311C), column compartment (G1316A), an automatic liquid sampler module (ALS, G7129A) and a Refractive Index Detector (G1362A, Agilent Technologies, Santa Clara, CA, USA).

The isocratic analysis was performed at 0.7 mL min^−1^ using 80% acetonitrile and 20% water [type I, 0.055 μS cm^−1^ at 25 °C, 5 μg L^−1^ TOC obtained using an A10 Milli-Q Advantage system and an Elix 35 (Merck KGaA, Darmstadt Germany)], 5 μL were injected into the system. We got a complete chromatographic run for glucose under 8 min using an amine-based chromatographic column for analytical separation (Zorbax Carbohydrate Analysis, 4.6 mm ID × 150 mm, 5 μm, Agilent Technologies, Santa Clara, CA, USA).

### Method optimization

**Fig. 1:**
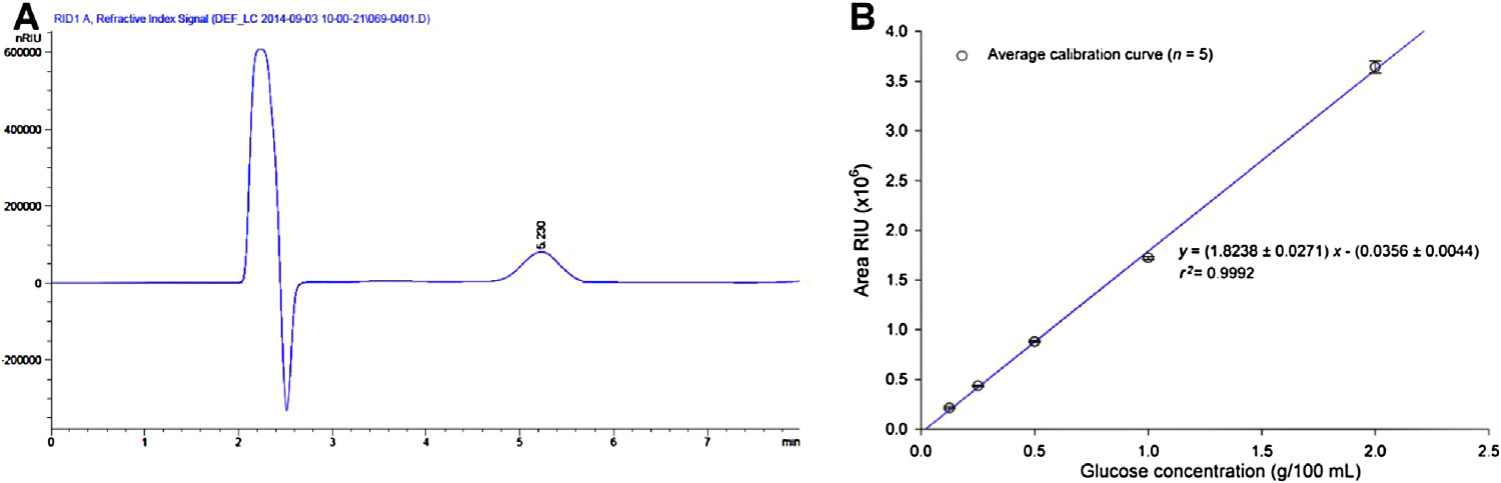
A. Chromatograph obtained using the proposed method, glucose_(*tr*=5.220)_ as a result of starch enzymatic degradation. B. The average calibration curve obtained from separate glucose standards, error bars denote repeatability.

The method was optimized using 1.5 g of feed mass; for sample 1327 masses from 0.22 to 0.98 g were tested, giving areas ranging from 5.4 × 10^5^ to 2.3 × 10^6^ which increase sensibility of the method and ease chromatographic integration. Similarly, we determined that our proposed procedure starch highest recovery was obtained when doubling enzyme units added to feed mixture concerning the reference method. When feed ingredients (e.g., ground corn) which contain substantial quantities of starch (i.e., over 45 g starch/100 g), sample initial mass must be reduced to 0.25 g to account for method’s dynamic range. On cases in which the glucose chromatographic signal (obtained after sample treatment, Fig. 1A) is deemed too low, injection volumes and sample mass can be modified to improve limit of detection or signal intensities.

### Method performance parameters

Five-point standard calibration curves were prepared using concentrations ranging from 0.125 to 2.50 g glucose/100 mL (and their respective areas under the curve). The resulting general equation derived from five different calibrations curves was: *y* = (1.824 ± 0.027)*x +* (0.036 ± 0.004) with an average coefficient of determination of 0.9992 (Fig. 1B). Sensibility (expressed as limits of detection and quantification, respectively) was calculated to be 0.02 g glucose/mL (0.09 g glucose/100 g feed) and 0.06 g glucose/mL (0.27 g glucose/100 g feed), which translates 0.25 g starch/100 g feed and 0.83 g starch/100 g feed, respectively. However, it is highly unlikely to encounter feed samples with starch values that low. On another hand, column efficiency was assessed as the number of theoretical plates (i.e., 860) and a height equivalent to a theoretical plate (i.e., 5.73). Peak asymmetry and tailing factor were determined which resulted in 1.20 (which represents a very slight peak fronting; normal for this column) and 1.11, respectively. The performance parameters are specific of the amino-based stationary phase column used during the assay, however columns governed by other chemical principles may be used to analyzed glucose (e.g., ion-exchange ligand-exchange columns). Additionally, AAFCO check sample program samples 1321, 1327, 1329, 1330, 1332 and 1421 were used as reference materials during validation of method accuracy, bias, and repeatability. Triplicates of samples 1325, 1332, 1329, and 1421 were used to assess method intra-day repeatability which ranged, expressed as RSD, from 0.22 to 1.98%. As expected, the sample with the lower starch value, exhibited more variability. Inter-day repeatability was measured using 7 replicates of sample 1330 which experimental starch values ranged from 21.55 to 25.96 and *z* values from −1.57 to 0.73. The method was able to assess starch values in different feed samples from 16.72 to 40.20 g/100 g with *z* values −1.99 to 0.43. Results obtained from LGC Standards proficiency scheme during 2015 and 2016 (AFPS 21 and 25, respectively) demonstrate the applicability of the method (i.e., *z* values ranging from −0.48 to 0.17, Table 1). Additionally, several local feed ingredients and compound feed for livestock on different growth stages (i.e., cattle and swine) were tested with the method above (Table 2). Results for distiller's dried grains with solubles [brewers’ dried grains, *n* = 15, (4.69 ± 0.68) g/100 g], rice bran [*n* = 8, (27.23 ± 1.26) g/100 g] and wheat middlings [*n* = 14, (27.16 ± 2.15) g/100 g] corn meal [*n* = 15, (70.36 ± 4.81) g/100 g] were in line with those reported elsewhere (Table 2). This data exemplifies the range and flexibility of the method.

**Table 1:** Results obtained by the suggested method for starch on different animal feeds and a feed ingredient.

Sample ID	Matrix	Experimental glucose mean value ± SD	Experimental starch mean value ± SD	Starch robust mean (g/100 g)	Starch robust SD (g/100 g)	*z* value
*AAFCO Check Sample Program – Animal Feed Scheme*						
1321	Dry Dog Food	13.82 ± 0.24	37.30 ± 1.71	40.20	1.92	−1.51
1327	Corn Gluten Meal	5.99 ± 0.12	16.16 ± 1.98	17.62	0.73	−1.99
1329	Calf Starter/Grower, Medicated	11.27 ± 0.08	30.42 ± 0.69	29.27	2.70	0.43
1330	Chick Starter, Medicated	8.10 ± 0.12	21.88 ± 1.51	23.95	1.53	−1.35
1332	Show Pig Finisher, Medicated	15.42 ± 0.06	41.63 ± 0.41	39.78	2.54	0.73
1421	Ewe Developer & Gestation Feed, Medicated	5.44 ± 0.10	14.68 ± 0.88	16.58	1.79	−1.06

*LGC Standards AFPS – Animal Feeds PT Scheme*						
AFPS 21	Chicken laying feed	8.03 ± 0.05	32.76 ± 0.58	32.20	0.21	−0.48^a^
AFPS 25	Cattle feed	12.16 ± 0.03	21.64 ± 0.22	20.59	3.87	0.17^a^

a z’ value calculated instead of z value considering method reported uncertainty value.

**Table 2:** Experimental starch values obtained for commercial feed ingredients and compound feed using the proposed method.

Matrix/parameter	Mean ± SD	Median	Max	Min	Reference/guaranteed values
	*Concentration,* g*/100* g				
*Feed ingredients*					
Distiller's Dried Grains with Solubles (*n* = 15)	4.69 ± 0.68	4.61	6.18	3.45	4.23 ± 1.4 [8]
Rice Bran, full fat (*n* = 8)	27.23 ± 1.26	27.02	29.28	25.26	27.4 ± 7.1 [12]
Wheat middlings (*n* = 14)	27.16 ± 2.15	27.00	30.63	23.94	27.7 ± 5.6 [12]
Corn meal (*n* = 15)	70.36 ± 4.81	71.30	77.42	61.20	73.2 ± 4.4 [18]
Alfalfa pelletized (*n* = 1)	2.13 ± 0.00				0.0 ± 0.0 [12]
Oat groats (*n* = 1)	46.18 ± 0.00				52.6 ± 1.6 [12]

*Compound feed*					
Equine feed (*n* = 11)	36.62 ± 10.73	34.34	56.87	22.92	
Hen feed (*n* = 11)	30.94 ± 5.53	31.24	38.77	23.59	
Poultry feed, starter (*n* = 7)	33.97 ± 8.59	30.99	46.38	21.58	
Poultry feed, grower (*n* = 7)	32.72 ± 7.57	28.46	44.19	25.67	
Poultry feed, finisher (*n* = 5)	30.72 ± 4.36	28.22	38.05	26.65	
Calf feed (*n* = 4)	38.01 ± 4.91	37.12	45.72	32.09	
Cattle feed (*n* = 14)	28.71 ± 8.48	25.79	39.45	10.50	
Dairy cattle (*n* = 12)	26.30 ± 1.75	26.36	28.95	21.71	
Pig, grower (*n* = 8)	32.19 ± 3.02	31.21	37.60	28.84	
Pig, finisher (*n* = 4)	31.61 ± 4.29	31.38	36.62	27.05	
Gestating sow (*n* = 1)	37.67 ± 0.00				

### Silages

Silages present a singularity since the organic acids that are a result of the fermentation process suffered by the raw plant material generate solutions with low pH. The α-amylase will not work under these conditions. Hence, the preparation of the enzyme in the MOPS buffer, adjusted to pH 6.2, which is the optimum pH value for α-amylase assisted hydrolysis [13], circumvents this issue. A pineapple residue silage that was engineered with 45% inclusion of square banana was determined to exhibit (11.89 ± 2.58) g/100 g starch (Table 3). A tropical corn silage delivered values of (24.36 ± 2.08) g/100 g starch, a concentration in line with a normal fiber corn silage [14]. Both results including resistant starch and on dry matter basis.

**Table 3:** Total and digestible starch in selected feed ingredients.

Matrix/Starch measurement	Digestible	Total	Resistant
	*Concentration,* g*/100* g^a^^,^^b^		*Fraction*
Square banana meal, *Musa Balbisiana* Colla	51.01 ± 0.74	71.81 ± 1.05	0.41 ± 0.05
Cassava meal, *Manihot esculenta* Crantz	90.36 ± 1.09	92.10 ± 1.33	0.019 ± 0.03
Pineapple silage, *Ananas comosus* (L.) Merr.	0.99 ± 0.17	1.85 ± 0.17	0.54 ± 0.05
Pineapple and square banana (15 g/100 g) silage	3.83 ± 2.89	7.09 ± 2.89	0.53 ± 0.22
Pineapple and square banana (30 g/100 g) silage	5.26 ± 3.14	9.74 ± 3.14	0.52 ± 0.17
Pineapple and square banana (45 g/100 g) silage	6.42 ± 2.60	11.89 ± 2.58	0.52 ± 0.11

a Values obtained for *n* = 5 replicates and expressed as mean ± standard deviation (SD).

b Results on dry matter basis.

### Total versus resistant starch in feed ingredients

Method 996.11 considers the determination of resistant starch which includes an initial DMSO solubilization step. Initially developed for cereals, the method has found success in the determination of resistant starch fractions in other feed ingredients such as cassava or square banana meals (Table 3) or, as stated above, in silages. The resistant starch fraction found in cassava flour (0.019 ± 0.03 which represent 16.74 g/kg) this concurs with data reported previously [15] ranging from 0.19 to 2.21 g/100 g. Total starch for the square banana meal was calculated to be (71.81 ± 1.05) g/100 g; starch is considered to be the major constituent in unripe green banana [16] resistant starch percentages as high as 54% have been reported for square banana [17].

### Calculations

Total starch is calculated as follows:

$$Estimated\;glucose\;\left(\frac{g}{100\;mL}\right)\cdot 10\;mL=Total\;glucose\;\left(g\right)$$

$$\frac{Total\;glucose\;\left(g\right)}{Sample\;mass\;\left(g\right)}\cdot \frac{81}{30}\cdot 100=Total\;starch\;\left(g/100\;g\right)$$
